# High Prevalence of Highly Pathogenic Avian Influenza: A Virus in Vietnam's Live Bird Markets

**DOI:** 10.1093/ofid/ofae355

**Published:** 2024-07-11

**Authors:** Duy Tung Dao, Kristen K Coleman, Vuong N Bui, Anh N Bui, Long H Tran, Quy D Nguyen, Son Than, Laura A Pulscher, Lyudmyla V Marushchak, Emily R Robie, Hung Nguyen-Viet, Phuc Duc Pham, Nathaniel C Christy, John S Brooks, Huy C Nguyen, Adam M Rubrum, Richard J Webby, Gregory C Gray

**Affiliations:** Virology Department, National Institute of Veterinary Research, Hanoi, Vietnam; Programme in Emerging Infectious Diseases, Duke-NUS Medical School, National University of Singapore, Singapore, Singapore; Department of Global, Environmental, and Occupational Health, University of Maryland School of Public Health, College Park, Maryland, USA; Department of Veterinary Medicine, College of Agriculture and Natural Resources, University of Maryland, College Park, Maryland, USA; Virology Department, National Institute of Veterinary Research, Hanoi, Vietnam; Virology Department, National Institute of Veterinary Research, Hanoi, Vietnam; Virology Department, National Institute of Veterinary Research, Hanoi, Vietnam; Virology Department, National Institute of Veterinary Research, Hanoi, Vietnam; Virology Department, National Institute of Veterinary Research, Hanoi, Vietnam; Programme in Emerging Infectious Diseases, Duke-NUS Medical School, National University of Singapore, Singapore, Singapore; Division of Infectious Diseases, Department of Medicine, University of Texas Medical Branch, Galveston, Texas, USA; Division of Infectious Diseases, Department of Medicine, University of Texas Medical Branch, Galveston, Texas, USA; Global Health Institute, Duke University, Durham, North Carolina, USA; International Livestock Research Institute, Hanoi, Vietnam; Center for Public Health and Ecosystem Research, Hanoi University of Public Health, Hanoi, Vietnam; U.S. Naval Medical Research Unit INDO PACIFIC, Singapore, Singapore; U.S. Naval Medical Research Unit INDO PACIFIC, Singapore, Singapore; U.S. Naval Medical Research Unit INDO PACIFIC, Singapore, Singapore; Department of Host-Microbe Interactions, St. Jude Children's Research Hospital, Memphis, Tennessee, USA; Department of Host-Microbe Interactions, St. Jude Children's Research Hospital, Memphis, Tennessee, USA; Division of Infectious Diseases, Department of Medicine, University of Texas Medical Branch, Galveston, Texas, USA; Department of Microbiology and Immunology, University of Texas Medical Branch, Galveston, Texas, USA; Institute for Human Infections and Immunity, University of Texas Medical Branch, Galveston, Texas, USA; Department of Global Health, School of Public and Population Health, University of Texas Medical Branch, Galveston, Texas, USA

**Keywords:** avian influenza virus, highly pathogenic avian influenza virus, influenza virus, zoonosis, zoonotic influenza

## Abstract

**Background:**

In recent years, Vietnam has suffered multiple epizootics of influenza in poultry.

**Methods:**

From 10 January 2019 to 26 April 2021, we employed a One Health influenza surveillance approach at live bird markets (LBMs) and swine farms in Northern Vietnam. When the COVID-19 pandemic permitted, each month, field teams collected oral secretion samples from poultry and pigs, animal facility bioaerosol and fecal samples, and animal worker nasal washes at 4 LBMs and 5 swine farms across 5 sites. Initially samples were screened with molecular assays followed by culture in embryonated eggs (poultry swabs) or Madin-Darby canine kidney cells (human or swine swabs).

**Results:**

Many of the 3493 samples collected had either molecular or culture evidence for influenza A virus, including 314 (37.5%) of the 837 poultry oropharyngeal swabs, 144 (25.1%) of the 574 bioaerosol samples, 438 (34.9%) of the 1257 poultry fecal swab samples, and 16 (1.9%) of the 828 human nasal washes. Culturing poultry samples yielded 454 influenza A isolates, 83 of which were H5, and 70 (84.3%) of these were highly pathogenic. Additionally, a positive human sample had a H9N2 avian-like PB1 gene. In contrast, the prevalence of influenza A in the swine farms was much lower with only 6 (0.4%) of the 1700 total swine farm samples studied, having molecular evidence for influenza A virus.

**Conclusions:**

This study suggests that Vietnam's LBMs continue to harbor high prevalences of avian influenza A viruses, including many highly pathogenic H5N6 strains, which will continue to threaten poultry and humans.

## Teaser

Live bird markets in Vietnam continue to harbor highly pathogenic avian influenza viruses that threaten poultry and human populations within and outside of the geographical region. Developed nations need to find ways to partner with Vietnam to reduce these threats.

Avian influenza viruses have historically caused large numbers of poultry deaths as well as a wide spectrum of illness in humans ranging from mild respiratory symptoms to severe disease that resulted in death. Novel influenza A viruses have often been first detected in North Asia and spread to other countries through migrating birds or movement of poultry across borders [1–3]. Vietnam has repeatedly suffered from these cross-border transmission events [4], leading to a higher risk of dangerous human exposure to novel influenza viruses and causing significant economic harm when the culling of agricultural flocks and herds has been necessary as a control measure. This human-animal interface environment remains poorly defined but can be expected to continue to have significant impacts on global health security and economic stability, as the COVID-19 pandemic has made abundantly clear. With a goal of improving surveillance for influenza A viruses, we sought to combine bioaerosol and animal worker sampling with traditional poultry and swine sampling methods at a number of sites at the northern Vietnam border.

## METHODS

### Study Sites

**Figure 1.**
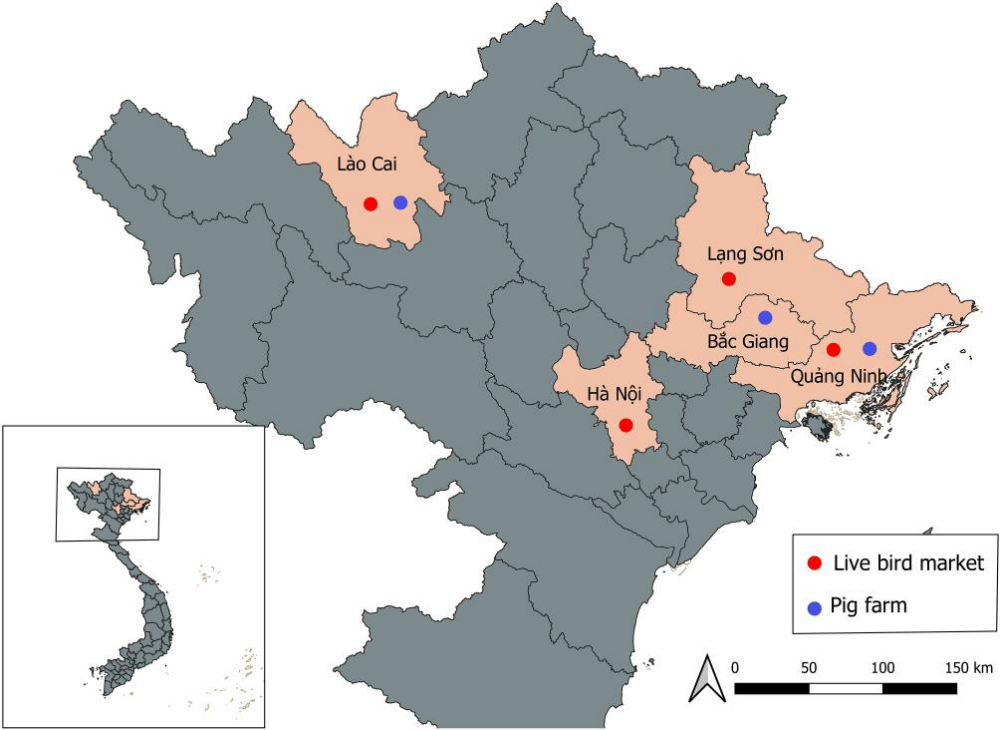
Approximate geographical locations of live bird markets and swine farms studied in Northern Vietnam, 10 January 2019 to 26 April 2021.

Environmental, animal, and animal worker specimens were collected from 4 live bird markets (LBM) and 5 swine farms in 5 sites (Hanoi City, Lang Son Province, Lao Cai Province, Bac Giang Province, and Quang Ninh Province) in Northern Vietnam (Figure 1). Study team members performed monthly sampling visits at LBMs and swine farms selected according to an agreement between the National Institute of Veterinary Research (NIVR) and Sub-Department of Animal Health in each province.

### Sample Collection

#### Live Bird Markets

Following techniques we had previously used in LBMs in Vietnam [5], China [6], Malaysia [7], and Myanmar [8], during each sampling visit, up to 3 National Institute for Occupational Safety and Health (NIOSH) 2-stage aerosol samplers were assembled on a tripod and positioned 0.5 m from the ground in poultry-dense sections of the market. Samplers were outfitted with a 15-mL Falcon tube, 1.5-mL centrifuge tube, and a polytetrafluoroethylene filter designed to collect viral particles. SKC AirChek Touch pumps (SKC, Eighty Four, Pennsylvania) were connected to the NIOSH samplers and run for 4 hours collecting air at 3.5 L/min.

Each visit, up to 10 oropharyngeal and 15 environmental fecal swabs were collected from poultry found near the NIOSH aerosol samplers, with preferential selection for any birds that appeared sick. Swabs were placed individually into sterile tubes containing 2.5 mL of viral transport medium.

Market poultry workers provided informed consent for the collection of 1 nasal wash. Participants were directed to tilt their heads back at a 70-degree angle, holding their breath as 1 nostril was irrigated with a teaspoon (5 mL) of sterile water using a 10-mL plastic syringe. The participant then tilted their head back down, releasing the fluid into a sterile specimen collection cup. These workers were additionally asked to complete a brief questionnaire regarding potential exposure to and knowledge of avian influenzas. Following initial study enrollment, workers were able to participate during subsequent visits.

#### Swine Farms

During each sampling visit, bioaerosol samples were collected near swine enclosures using NIOSH samplers, as described previously. For each bioaerosol sampler, 3 pig oral secretions and 3 environmental fecal swabs were gathered from the immediate area.

Pig oral secretions were collected by fixing a sterile water-wetted piece of cotton rope to the swine enclosure at the approximate head height of the pigs. Animals were then allowed to chew on the rope for 30–45 minutes, after which the trapped liquid was expressed into a 2-mL cryovial. Environmental fecal swabs were collected from within pig enclosures and stored individually in tubes containing 2.5 mL of viral transport media.

At each site visit, farm workers with occupational exposure to swine provided informed consent for the collection of 1 nasal wash, as previously described, and a serum sample. Workers were asked to complete a brief questionnaire regarding knowledge and practices surrounding zoonotic influenza transmission. Participants previously enrolled in the study were additionally asked to provide one 5-mL venous blood sample during subsequent sampling times.

### Sample Processing

All samples were stored on ice for transfer to the central laboratory at NIVR in Hanoi, Vietnam. On arrival, samples were either processed immediately or preserved at −80 °C. Sera were separated and archived −80 °C for future seroepidemiology assessments.

#### Bioaerosol Samples

NIOSH samplers were disassembled in the laboratory for immediate processing. Sterile virus collection medium (phosphate buffered saline with bovine serum albumin fraction V) were added to both the 15-mL Falcon tube and 1.5-mL centrifuge tube, after which each was vortexed thoroughly before transferring the liquid wash to individual 2.0-mL cryovial tubes. The polytetrafluoroethylene filter was then removed from its casing and dry vortexed for 15 seconds in a 50-mL Falcon tube. Next, virus collection media was added to the tube containing the filter, taking care to wet the filter. After thorough vortexing, the filter was discarded, and the remaining media was combined with the specimen collected in the cryovial from the 1.5-mL centrifuge tube.

#### Swab Samples and Nasal Washes

Tubes containing collected swabs were vortexed at medium speed. Swabs were then discarded, and the remaining viral transport media was transferred to 2.0-mL cryovials. Worker nasal wash and pig oral secretion samples required no additional processing before molecular work.

#### Laboratory Studies

Initially samples were screened with molecular assays followed by culture in embryonated eggs (poultry swabs) or Madin-Darby canine kidney cells (human or swine swabs) at NIVR, Hanoi, Vietnam. Later, to save funds, field specimens were directly inoculated into eggs. Poultry, swine samples, and human samples that had culture or molecular evidence of influenza A virus were then transported on dry ice to the University of Texas Medical Branch (UTMB), Galveston, TX, USA, for further characterization.

### Molecular Analysis

At NIVR, viral RNA was extracted from processed specimen using the QIAamp Viral RNA Mini Kit (Qiagen). Sample RNA was screened for influenza A virus using quantitative real-time reverse transcription polymerase chain reaction (qRT-PCR) as described by the World Health Organization (WHO) [9].

To further characterize positive specimens at UTMB, poultry and poultry environmental cultures and swine oral secretion cultures were inactivated in TRIzol LS Reagent (Invitrogen, Waltham, MA) under BSL3E conditions before being moved into BSL2E where they underwent RNA extraction following the manufacturer's recommendations (TRIzol LS Reagent, Pub. No. MAN0000806 Rev. B.0) and then stored at −80 °C until further analysis. Poultry specimens were studied with qRT-PCR for H5 and H7 based on WHO recommended protocols for Eurasian avian influenza virus subtypes [10, 11]. H5-positive samples were further characterized to differentiate high pathogenic avian influenza virus from low pathogenic avian influenza virus subtypes by an RT-PCR targeting the HA0 motif for amplicon sequencing [12]. Additionally, a 10% subset (n = 45) of poultry cultures was studied for H9 by qRT-PCR based on WHO recommended protocols [10]. H9-positive samples with high viral load (Ct ≤ 15) were further characterized targeting the hemagglutinin (HA) gene of H9 subtypes by conventional RT-PCR for amplicon sequencing [13].

For influenza A virus positive human nasal wash samples, RNA was extracted using a QIAamp Viral RNA mini kit (Qiagen). Samples were first studied with qRT-PCR for seasonal human H1N1 pandemic and H3N2 subtypes using WHO-recommended protocols. Samples that had molecular evidence of influenza A matrix gene were then further studied using a universal primer set to target the HA and NA genes for amplicon sequencing [14].

Additionally, a subset (n = 20) of influenza A-positive samples from poultry, swine, and humans were sent for next-generation sequencing (NGS) at St. Jude Children's Research Hospital using a universal primer set for amplicon sequencing [15]. Samples were chosen for NGS based on Ct value, with low Ct values having priority, and overall diversity across years, sample type, and location as well as diversity across the HA0 motif gene. All amplicons were sent for Illumina sequencing and assembled using CLC Genomics workbench (v.21.0.5). Assembled sequences of the HA and NA genes were compared against the nucleotide database of the National Center for Biotechnology Information by using Basic Local Alignment Search Tool (NCBI Blast). Phylogenetic analysis was performed using Geneious Prime software V 2023.2.1 (Dotmatics, Boston, MA) employing the Tamura-Nei model genetic distance values.

### Patient Consent Statement

The authors confirm that written consent was obtained from all participants. The study protocol was approved by the institutional review boards at University of Texas Medical Branch, Duke University, Duke-NUS Medical School, and Hanoi University of Public Health, Hanoi, Vietnam, in compliance with all applicable federal regulations governing the protection of human subjects. The study protocol was also reviewed by the US Department of the Navy Human Research Protection Program officials. Institutional Animal Care and Use Committee approval was granted by University of Texas Medical Branch and Duke University in compliance with all applicable federal regulations governing the protection of animals in research.

## RESULTS

The prevalence of influenza A was high at LBMs (Table 1). Of the poultry and poultry environmental samples tested, many had either molecular or culture evidence for influenza A virus, with the highest prevalence in poultry oropharyngeal swabs (37.5%, n = 314), followed by poultry fecal swabs (34.9%, n = 438) and bioaerosol samples (25.1%, n = 144). By comparison, only 16 (1.9%) of the 828 human nasal washes had evidence of influenza A virus. Of the 454 poultry or poultry environmental isolates characterized at UTMB, 83 (18.3%) were positive for H5 and the majority of these (84.3%, n = 70) were determined to be highly pathogenic avian influenza H5 viruses by sanger sequencing (Supplementary Table 1). The remaining 13 isolates were undetermined. Additionally, of the poultry fecal isolates (n = 45) tested for H9 at UTMB by qRT-PCR, 43 (96.0%) were positive. A small subset (n = 13) of H9-positive samples were sent for Sanger sequencing and partial sequences were most closely aligned to HA genes from H9N2 viruses. None of the poultry samples tested positive for H7 subtypes. We have not yet studied swine worker sera.

**Table 1.** Summary of Field and Laboratory Results From Live Bird Market (LBM) and Swine Farm Samples Collected in Northern Vietnam From 10 January 2019 to 26 April 2021

Sample Types	Number Collected	Molecular Screening			Cell or Egg Culture Screening			Molecular and Cell or Egg Culture Screening	
		Number of Specimens Examined with qRT-PCR	Number of Specimens Positive for Influenza A by qRT-PCR	Among Specimens Studied, Percent Positive for Influenza A Virus by qRT-PCR	Number of Specimens Examined With Cell or Egg Culture	Number of Specimens to Yield Influenza A Virus From Cell or Egg Culture	Percent to Yield Influenza A Virus From Cell or Egg Culture	Number of Specimens Positive for Either qRT-PCR or Culture	Percent of Specimens Positive for Either qRT-PCR or Culture
LBM human nasal washes	828	828	16	1.9%	575	0	0.0%	16	1.9%
LBM poultry oropharyngeal swabs	837	837	267	31.9%	815	229	28.1%	314	37.5%
LBM poultry cage swabs	1257	1255	327	26.1%	1218	302	24.8%	438	34.9%
LBM bioaerosols	574	573	124	21.6%	539	51	9.5%	144	25.1%
Swine human nasal washes	370	339	0	0.0%	370	0	0.0%	0	0.0%
Swine oral secretions	570	506	6	1.2%	570	0	0.0%	6	1.1%
Swine fecal slurry	571	523	0	0.0%	571	0	0.0%	0	0.0%
Swine farm bioaerosols	191	175	0	0.0%	191	0	0.0%	0	0.0%

Abbreviation: qRT-PCR, quantitative real-time reverse transcriptase polymerase chain reaction.

A subset of 20 H5-positive poultry or poultry environmental samples were submitted for NGS sequencing. Of the samples characterized by NGS, a variety of subtypes were identified, with H5N6 (n = 11) being the predominant subtype identified, followed by H9N2 (n = 3), H5N2 (n = 2), H3N2 (n = 1), and H6N6 (n = 1). Additionally, 1 sample had a mixture of subtypes H5N6 and H3N2 and 1 sample was partially characterized as HxN2. GenBank sequence submission data can be viewed in the supplemental material.

**Figure 2.**
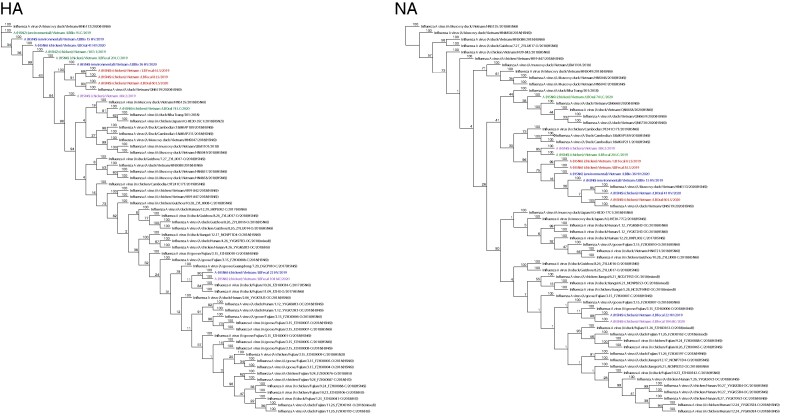
A neighbor-joining phylogenetic tree of the hemagglutinin (HA) gene segments of the isolated poultry H5N6 and H5N2 viruses and neuraminidase (NA) segments of isolated poultry H5N6 viruses. Red = HA and NA sequences from specimens collected in the Lang Son Province, Vietnam; green = HA and NA sequences from specimens collected in the Lao Cai Province, Vietnam; blue = HA and NA sequences from specimens collected in Hanoi, Vietnam; purple = HA and NA sequences from specimens collected in Quang Ninh Province, Vietnam; black = representative viruses from GenBank.

**Figure 3.**
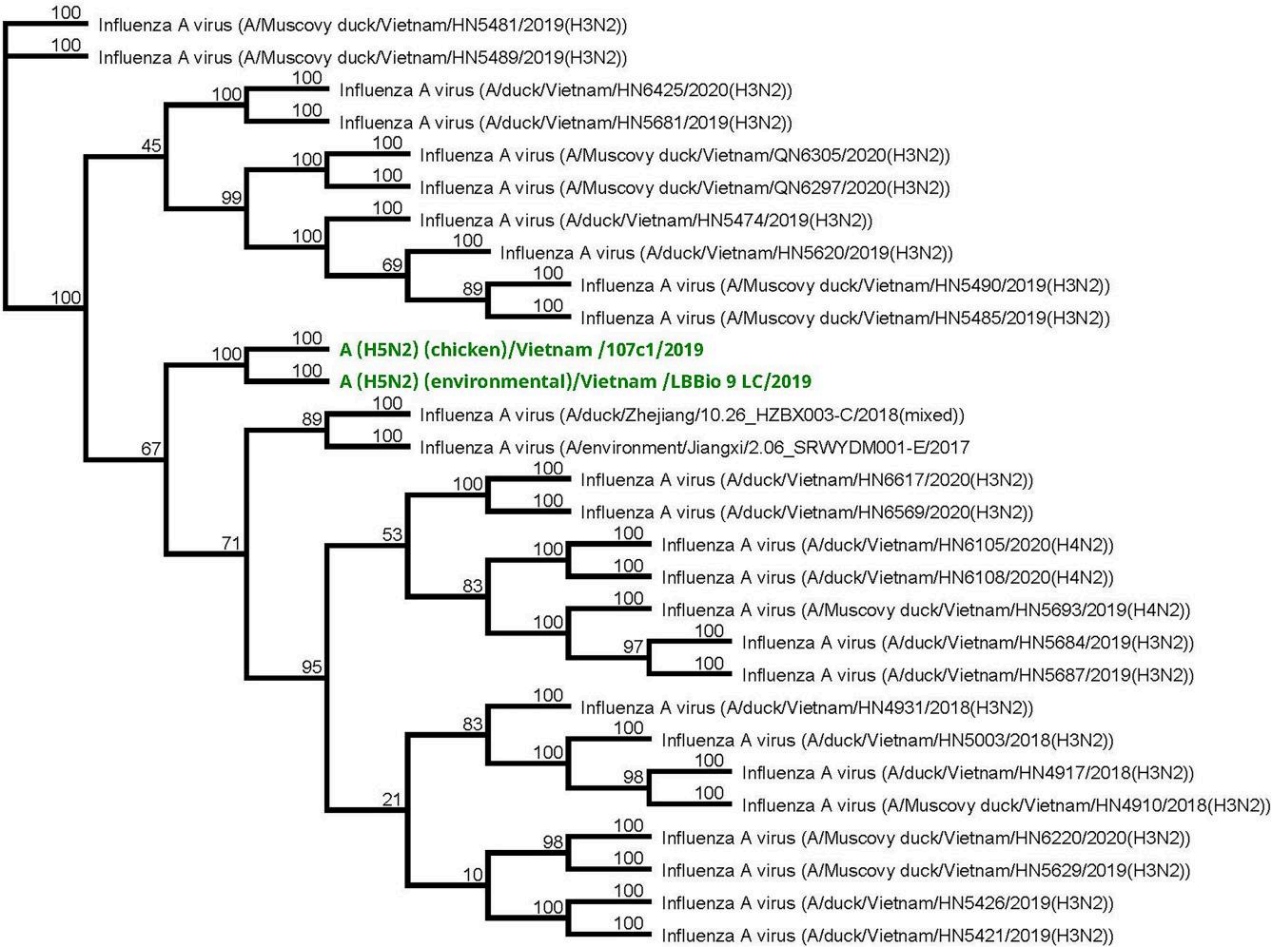
A neighbor-joining phylogenetic tree of the neuraminidase (NA)(N2) gene segments of the isolated H5N2 poultry viruses collected in Lao Cai Province, Vietnam, compared to other representative HxN2 viruses from GenBank.

Phylogenetic analysis of the H5N6 and H5N2 specimens resulted in a wide variety of influenza A viruses with 3 different clusters identified for the HA (H5) gene and 3 tree clusters identified for the NA (N6) gene (Figures 2 and 3). Most of the H5N6 and the H5N2 samples clustered closely with the HA and NA genes of Vietnamese Muscovy ducks, but 1 cluster was closely aligned with samples collected from Chinese poultry (Figures 2 and 3). The H5 sequences for both H5N6 and H5N2 viruses had a PLRE RRRKR GLR cleavage motif between HA1 and HA2 confirming our Sanger sequencing results that these are highly pathogenic avian influenza viruses belonging to clade 2.3.4.

**Figure 4.**
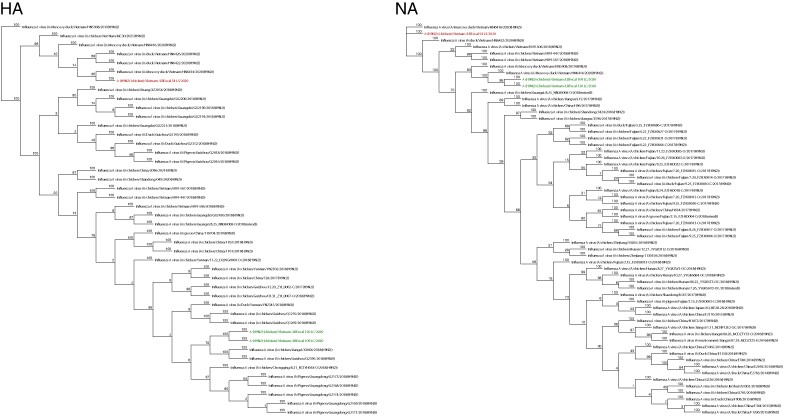
A neighbor-joining phylogenetic tree of the hemagglutinin (HA) and neuraminidase (NA) gene segments of the isolated poultry H9N2 viruses. Red = HA and NA sequences from specimens collected in Lang Son Province, Vietnam; green = HA and NA sequences from specimens collected in Lao Cai Province, Vietnam; black = representative H9N2 viruses from GenBank.

Of the H9N2 isolates characterized through NGS, all shared close identity with H9N2 collected from poultry in Vietnam or China, which confirmed our Sanger sequencing results (Figure 4). The H3N2 and H6N6 isolates also shared close identity with poultry samples previously collected from Vietnamese or Chinese ducks (Supplementary Figs. 1, 2, 3 and 4).

Of the 16 LBM human nasal washes that were positive for the influenza A matrix gene, none was positive for seasonal human influenza viruses H1N1 pandemic or H3N2. An RT-PCR targeting the universal genes of influenza A viruses was attempted on all 16 human nasal washes and 1 sample returned good partial Sanger sequence results for the PB1 gene. Phylogenetic analysis showed this PB1 gene sequence clustered with sequences from H9N2 avian influenza A viruses identified from domestic Muscovy ducks in Vietnam, as well as a chicken from Japan (Supplementary Fig. 5). Sequences could not be obtained for HA or NA genes for this or any of the other human samples and, although NGS sequencing was attempted on this sample, Ct values were high (all had Ct values >30) and whole genome sequences could not be obtained.

In contrast to the LBMs, the prevalence for influenza A at the swine farms was much lower, with only 6 (0.35%) of the 1700 total swine farm samples studied having molecular evidence for influenza A virus (Table 1). All 6 were swine oropharyngeal samples collected from a farm in Hiep Hoa district in Bac Giang Province during 2020. Four swine samples yielded full genomes and were characterized as H1N1. One additional sample had an HA that could not be determined but the NA was identified as N1. Phylogenetic analysis of samples with good sequences revealed HA (H1) genes clustered most closely with swine isolates collected from China (Supplementary Fig. 6).

## DISCUSSION

The main objective of this study was to conduct surveillance for influenza A viruses among LBMs and swine farms in Northern Vietnam, with a specific focus on possible novel virus introductions from China. Evidence for such China-to-Vietnam influenza virus incursions have been reported for avian H5N1, H5N6, H6, and H9 strains [16–18]. Because avian influenza was known to be prevalent among Vietnamese poultry before our study, further novel virus introductions from China, such as H7N9 strains, could mix with Vietnamese strains and threaten poultry, yielding additional novel and virulent strains that might further threaten Vietnamese poultry and humans. Hence, we sampled geographical areas where viruses from China might enter Vietnamese agriculture. Fortunately, we did not find evidence of H7N9 or other viral incursions from China.

Even so, we compared to our previous studies [5, 6, 19–22], the LBM sites had surprisingly high prevalence estimates for influenza A positivity, and notably for highly pathogenic H5 subtypes. These findings were not totally surprising as highly pathogenic H5 subtypes have been circulating in Vietnam for approximately 20 years [23]. Interestingly, some of the H5-positive samples submitted for NGS identified subtypes other than H5. Although the reason for the discrepancy between the qRT-PCR and NGS results is not fully known, such discrepancies have previously been noted and may be due to quality of the RNA or coverage and depth of the NGS reads [24, 25]. Our surveillance findings were not reported to be associated with unusual morbidity in LBMs, suggesting that high HPAI prevalence in Vietnamese LBM maybe normative.

Through this surveillance, we also demonstrated that small teams of field workers are capable of conducting monthly sampling visits using a comprehensive One Health approach that could provide local and national public health and domestic animal production officials’ rapid feedback regarding “hot spot” locations and animal specimen types with concerningly high rates of influenza A positivity.

In contrast to our LBM findings, the influenza A prevalence among swine farms was surprisingly low compared to the other swine farms studies we have conducted in other countries [26–29]. One wonders if this low prevalence is nationwide. Recently, a longitudinal study of swine influenza viruses from 23 areas of Vietnam obtained from slaughterhouse swine during 2010–2019 would support such a nationwide lower nationwide prevalence (mean, 1.95%; range, 0.33%–11.48%) [30]. Although one might argue that this study would naturally have a lower prevalence of influenza compared to our sometime sick pigs because they only sampled healthy adult pigs at their entrance into a abattoir. Alternatively, the low prevalence we found could simply indicate better national biosecurity after Vietnamese swine farms have recently suffered from multiple African swine fever incursions [31].

Our study additionally demonstrated that poultry fecal cage swabs and bioaerosols at LBMs are environmental sources of infectious influenza A viruses that may pose an occupational risk for zoonotic infection as evidenced by PCR-positive human nasal washes. Although the prevalence of influenza A virus was low among human nasal washes, a partial sequence of an H9N2 avian-like PB1 gene was identified in 1 human nasal wash, suggesting spillover of avian influenza A viruses may be occurring. Although we cannot ascertain what the influenza subtype was, a previous longitudinal cohort study found a 9% seroconversion rate to avian influenza H9 among Vietnamese farming households [32], suggesting infections among LBM workers may be occurring. Indeed, molecular and serological evidence of avian influenza H9N2 among poultry workers has been previously reported [33]. Furthermore, bioaerosol sampling appears to be a sufficient influenza virus surveillance tool that could potentially replace more invasive bird swab sampling. Such environmental sampling is relatively easy to use, highly portable, nonintrusive, and generally well accepted by market workers, making this surveillance tool a great alternative to the traditional methods of individual sampling of animals. Results from this study can inform future avian influenza surveillance and control efforts in rural and remote locations similar to the sampling sites in this study.

## Supplementary Material

ofae355_Supplementary_Data

## Supplementary Data

Supplementary materials are available at *Open Forum Infectious Diseases* online. Consisting of data provided by the authors to benefit the reader, the posted materials are not copyedited and are the sole responsibility of the authors, so questions or comments should be addressed to the corresponding author.

## Notes

***Acknowledgments.*** We thank Hisham Alsharif, Samantha Cody, Odmaa Ganzorig, Nathaniel Giles, Eelin Khurelbaatar, and Teresa Peret for their assistance om performing laboratory assay work at UTMB in support of this research.

***Financial support***. The study was funded by the United States Global Emerging Infections Surveillance (GEIS) program under AFHSD-Biosurveillance Branch (Armed Forces Health Surveillance Division) ProMIS ID number P0154_22_N2 and from Professor Gray's discretionary funding at the University of Texas Medical Branch. The views expressed in this article are those of the authors and should not be construed to reflect the official policy or represent the positions of the US Department of the Navy, Department of Defense, nor the United States government. LCDR Nathaniel Christy, MSC, NAMRU-IP; CAPT John S. Brooks, MC, USN NAMRU-IP; and LT Huy C. Nguyen, MC, USN NAMRU-IP are military service members. This work was prepared as part of their official duties. Title 17 U.S.C. 105 provides that “copyright protection under this title is not available for any work of the United States Government.” Title 17 U.S.C. 101 defines a US government work as work prepared by a military service member or employee of the US government as part of that person's official duties.
