# Supplementary material for: High Prevalence of Highly Pathogenic Avian Influenza: A Virus in Vietnam's Live Bird Markets
Source: Open Forum Infect Dis. 2024 Jul 11;11(7):ofae355. doi: 10.1093/ofid/ofae355 (PMC11250224; doi:10.1093/ofid/ofae355)
Supplement: ofae355_Supplementary_Data [file ofae355_supplementary_data.zip › Sup Matl Highly Pathogenic Avian Influenza Vietnam ver7.docx]

**Supplementary Material**

**High Prevalence of Highly Pathogenic Avian Influenza A Virus**

**in Vietnam’s Live Bird Markets**

Duy Tung Dao,^1^ Kristen K. Coleman,^2,3^ Vuong N. Bui,^1^ Anh N. Bui,^1^ Long H. Tran,^1^ Quy D. Nguyen,^1^ Son Than,^1,2^ Laura A. Pulscher,^4^ Lyudmyla V. Marushchak, ^4^ Emily R. Robie,^5^ Hung Nguyen-Viet,^6^ Phuc Duc Pham,^7^ Nathaniel C. Christy,^8^ John S. Brooks,^8^ Huy C. Nguyen,^8^ Adam M. Rubrum,^9^ Richard J. Webby,^9^ and Gregory C. Gray^4,10-12^

**GenBank sequence submission data**

| **iDPCC**  **Submission ID** | **Sequence Identifier** | **Sample Identifier** | **Gene**  **Segment** | **GenBank**  **Accession Number** |
| --- | --- | --- | --- | --- |
| 2578 | LBBio15HV.PA | LBBio15HV | PA | PP751659 |
| 2578 | LBBio15HV.NS | LBBio15HV | NS | PP751660 |
| 2578 | LBBio15HV.PB1 | LBBio15HV | PB1 | PP751661 |
| 2578 | LBBio15HV.NP | LBBio15HV | NP | PP751662 |
| 2578 | LBBio15HV.PB2 | LBBio15HV | PB2 | PP751663 |
| 2578 | LBBio15HV.HA | LBBio15HV | HA | PP751664 |
| 2578 | LBBio15HV.MP | LBBio15HV | MP | PP751665 |
| 2578 | LBBio15HV.NA | LBBio15HV | NA | PP751666 |
| 2578 | LBBio36HV.NA | LBBio36HV | NA | PP751780 |
| 2578 | LBBio36HV.PB2 | LBBio36HV | PB2 | PP751781 |
| 2578 | LBBio36HV.HA | LBBio36HV | HA | PP751782 |
| 2578 | LBBio36HV.NS | LBBio36HV | NS | PP751783 |
| 2578 | LBBio36HV.PB1 | LBBio36HV | PB1 | PP751784 |
| 2578 | LBBio36HV.NP | LBBio36HV | NP | PP751785 |
| 2578 | LBBio36HV.MP | LBBio36HV | MP | PP751786 |
| 2578 | LBBio36HV.PA | LBBio36HV | PA | PP751787 |
| 2578 | LBFecal104MC.NS | LBFecal104MC | NS | PP751686 |
| 2578 | LBFecal104MC.PB1 | LBFecal104MC | PB1 | PP751687 |
| 2578 | LBFecal104MC.NP | LBFecal104MC | NP | PP751688 |
| 2578 | LBFecal104MC.HA | LBFecal104MC | HA | PP751689 |
| 2578 | LBFecal104MC.PA | LBFecal104MC | PA | PP751690 |
| 2578 | LBFecal104MC.NA | LBFecal104MC | NA | PP751691 |
| 2578 | LBFecal104MC.PB2 | LBFecal104MC | PB2 | PP751692 |
| 2578 | LBFecal104MC.MP | LBFecal104MC | MP | PP751693 |
| 2578 | LBFecal120LC.NP | LBFecal120LC | NP | PP751718 |
| 2578 | LBFecal120LC.PB1 | LBFecal120LC | PB1 | PP751719 |
| 2578 | LBFecal120LC.PA | LBFecal120LC | PA | PP751720 |
| 2578 | LBFecal120LC.MP | LBFecal120LC | MP | PP751721 |
| 2578 | LBFecal120LC.NS | LBFecal120LC | NS | PP751722 |
| 2578 | LBFecal120LC.HA | LBFecal120LC | HA | PP751723 |
| 2578 | LBFecal120LC.PB2 | LBFecal120LC | PB2 | PP751724 |
| 2578 | LBFecal120LC.NA | LBFecal120LC | NA | PP751725 |
| 2578 | LBFecal20LC.NA | LBFecal20LC | NA | PP751764 |
| 2578 | LBFecal20LC.NS | LBFecal20LC | NS | PP751765 |
| 2578 | LBFecal20LC.NP | LBFecal20LC | NP | PP751766 |
| 2578 | LBFecal20LC.PB1 | LBFecal20LC | PB1 | PP751767 |
| 2578 | LBFecal20LC.PB2 | LBFecal20LC | PB2 | PP751768 |
| 2578 | LBFecal20LC.MP | LBFecal20LC | MP | PP751769 |
| 2578 | LBFecal20LC.HA | LBFecal20LC | HA | PP751770 |
| 2578 | LBFecal20LC.PA | LBFecal20LC | PA | PP751771 |
| 2578 | LBFecal44C2.MP | LBFecal44C2 | MP | PP751772 |
| 2578 | LBFecal44C2.PA | LBFecal44C2 | PA | PP751773 |
| 2578 | LBFecal44C2.PB2 | LBFecal44C2 | PB2 | PP751774 |
| 2578 | LBFecal44C2.NA | LBFecal44C2 | NA | PP751775 |
| 2578 | LBFecal44C2.NP | LBFecal44C2 | NP | PP751776 |
| 2578 | LBFecal44C2.HA | LBFecal44C2 | HA | PP751777 |
| 2578 | LBFecal44C2.PB1 | LBFecal44C2 | PB1 | PP751778 |
| 2578 | LBFecal44C2.NS | LBFecal44C2 | NS | PP751779 |
| 2578 | LBFecal54LS.NP | LBFecal54LS | NP | PP751678 |
| 2578 | LBFecal54LS.PA | LBFecal54LS | PA | PP751679 |
| 2578 | LBFecal54LS.PB2 | LBFecal54LS | PB2 | PP751680 |
| 2578 | LBFecal54LS.NA | LBFecal54LS | NA | PP751681 |
| 2578 | LBFecal54LS.PB1 | LBFecal54LS | PB1 | PP751682 |
| 2578 | LBFecal54LS.NS | LBFecal54LS | NS | PP751683 |
| 2578 | LBFecal54LS.MP | LBFecal54LS | MP | PP751684 |
| 2578 | LBFecal54LS.HA | LBFecal54LS | HA | PP751685 |
| 2578 | LBFecal67MC.PB2 | LBFecal67MC | PB2 | PP751694 |
| 2578 | LBFecal67MC.PB1 | LBFecal67MC | PB1 | PP751695 |
| 2578 | LBFecal67MC.NA | LBFecal67MC | NA | PP751696 |
| 2578 | LBFecal67MC.HA | LBFecal67MC | HA | PP751697 |
| 2578 | LBFecal67MC.MP | LBFecal67MC | MP | PP751698 |
| 2578 | LBFecal67MC.NS | LBFecal67MC | NS | PP751699 |
| 2578 | LBFecal67MC.NP | LBFecal67MC | NP | PP751700 |
| 2578 | LBFecal67MC.PA | LBFecal67MC | PA | PP751701 |
| 2578 | LBFecal6LS.HA | LBFecal6LS | HA | PP751756 |
| 2578 | LBFecal6LS.PB2 | LBFecal6LS | PB2 | PP751757 |
| 2578 | LBFecal6LS.NA | LBFecal6LS | NA | PP751758 |
| 2578 | LBFecal6LS.PB1 | LBFecal6LS | PB1 | PP751759 |
| 2578 | LBFecal6LS.NP | LBFecal6LS | NP | PP751760 |
| 2578 | LBFecal6LS.MP | LBFecal6LS | MP | PP751761 |
| 2578 | LBFecal6LS.NS | LBFecal6LS | NS | PP751762 |
| 2578 | LBFecal6LS.PA | LBFecal6LS | PA | PP751763 |
| 2578 | LBFecal80C2.PB1 | LBFecal80C2 | PB1 | PP751710 |
| 2578 | LBFecal80C2.NS | LBFecal80C2 | NS | PP751711 |
| 2578 | LBFecal80C2.PA | LBFecal80C2 | PA | PP751712 |
| 2578 | LBFecal80C2.NP | LBFecal80C2 | NP | PP751713 |
| 2578 | LBFecal80C2.NA | LBFecal80C2 | NA | PP751714 |
| 2578 | LBFecal80C2.MP | LBFecal80C2 | MP | PP751715 |
| 2578 | LBFecal80C2.HA | LBFecal80C2 | HA | PP751716 |
| 2578 | LBFecal80C2.PB2 | LBFecal80C2 | PB2 | PP751717 |
| 2578 | LBFecal8LS.NP | LBFecal8LS | NP | PP751667 |
| 2578 | LBFecal8LS.MP | LBFecal8LS | MP | PP751668 |
| 2578 | LBFecal8LS.PA | LBFecal8LS | PA | PP751669 |
| 2578 | LBFecal8LS.NA | LBFecal8LS | NA | PP751670 |
| 2578 | LBFecal8LS.HA | LBFecal8LS | HA | PP751671 |
| 2578 | LBFecal8LS.PB1 | LBFecal8LS | PB1 | PP751672 |
| 2578 | LBFecal8LS.NS | LBFecal8LS | NS | PP751673 |
| 2578 | LBFecal8LS.PB2 | LBFecal8LS | PB2 | PP751674 |
| 2578 | LBOral41HV.NS | LBOral41HV | NS | PP751702 |
| 2578 | LBOral41HV.HA | LBOral41HV | HA | PP751703 |
| 2578 | LBOral41HV.PA | LBOral41HV | PA | PP751704 |
| 2578 | LBOral41HV.NP | LBOral41HV | NP | PP751705 |
| 2578 | LBOral41HV.NA | LBOral41HV | NA | PP751706 |
| 2578 | LBOral41HV.MP | LBOral41HV | MP | PP751707 |
| 2578 | LBOral41HV.PB2 | LBOral41HV | PB2 | PP751708 |
| 2578 | LBOral41HV.PB1 | LBOral41HV | PB1 | PP751709 |
| 2578 | LBOral74LC.MP | LBOral74LC | MP | PP751726 |
| 2578 | LBOral74LC.NS | LBOral74LC | NS | PP751727 |
| 2578 | LBOral74LC.PB2 | LBOral74LC | PB2 | PP751728 |
| 2578 | LBOral74LC.PB1 | LBOral74LC | PB1 | PP751729 |
| 2578 | LBOral74LC.NA | LBOral74LC | NA | PP751730 |
| 2578 | LBOral74LC.NP | LBOral74LC | NP | PP751731 |
| 2578 | LBOral74LC.PA | LBOral74LC | PA | PP751732 |
| 2578 | LBOral74LC.HA | LBOral74LC | HA | PP751733 |
| 2578 | SWOral140BG.MP | SWOral140BG | MP | PP751745 |
| 2578 | SWOral140BG.NA | SWOral140BG | NA | PP751746 |
| 2578 | SWOral140BG.HA | SWOral140BG | HA | PP751747 |
| 2578 | SWOral140BG.PB1 | SWOral140BG | PB1 | PP751748 |
| 2578 | SWOral140BG.PA | SWOral140BG | PA | PP751749 |
| 2578 | SWOral140BG.PB2 | SWOral140BG | PB2 | PP751750 |
| 2578 | SWOral140BG.NS | SWOral140BG | NS | PP751751 |
| 2578 | SWOral140BG.NP | SWOral140BG | NP | PP751752 |
| 2578 | SWOral141BG.NS | SWOral141BG | NS | PP751641 |
| 2578 | SWOral141BG.MP | SWOral141BG | MP | PP751642 |
| 2578 | SWOral141BG.NA | SWOral141BG | NA | PP751643 |
| 2578 | SWOral141BG.PB1 | SWOral141BG | PB1 | PP751644 |
| 2578 | SWOral141BG.HA | SWOral141BG | HA | PP751645 |
| 2578 | SWOral141BG.PA | SWOral141BG | PA | PP751646 |
| 2578 | SWOral141BG.PB2 | SWOral141BG | PB2 | PP751647 |
| 2578 | SWOral141BG.NP | SWOral141BG | NP | PP751648 |
| 2578 | SWOral142BG.PA | SWOral142BG | PA | PP751649 |
| 2578 | SWOral142BG.NP | SWOral142BG | NP | PP751650 |
| 2578 | SWOral142BG.PB1 | SWOral142BG | PB1 | PP751651 |
| 2578 | SWOral142BG.NS | SWOral142BG | NS | PP751652 |
| 2578 | SWOral142BG.HA | SWOral142BG | HA | PP751653 |
| 2578 | SWOral142BG.PB2 | SWOral142BG | PB2 | PP751654 |
| 2578 | SWOral142BG.MP | SWOral142BG | MP | PP751655 |
| 2578 | SWOral142BG.NA | SWOral142BG | NA | PP751656 |
| 2578 | SWOral144BG.NP | SWOral144BG | NP | PP751734 |
| 2578 | SWOral144BG.PA | SWOral144BG | PA | PP751735 |
| 2578 | SWOral144BG.NS | SWOral144BG | NS | PP751736 |
| 2578 | SWOral144BG.PB2 | SWOral144BG | PB2 | PP751737 |
| 2578 | SWOral144BG.NA | SWOral144BG | NA | PP751738 |
| 2578 | SWOral144BG.HA | SWOral144BG | HA | PP751739 |
| 2578 | SWOral144BG.MP | SWOral144BG | MP | PP751740 |
| 2578 | SWOral144BG.PB1 | SWOral144BG | PB1 | PP751741 |
| 2578 | LBOral60LS.HA | LBOral60LS | HA | PP752015 |
| 2578 | LBOral60LS.NS | LBOral60LS | NS | PP752016 |
| 2578 | LBOral60LS.PB1 | LBOral60LS | PB1 | PP752017 |
| 2578 | LBOral60LS.MP | LBOral60LS | MP | PP752018 |
| 2578 | LBOral60LS.NP | LBOral60LS | NP | PP752019 |
| 2578 | LBOral60LS.NA | LBOral60LS | NA | PP752020 |
| 2578 | LBOral60LS.PB2 | LBOral60LS | PB2 | PP752021 |
| 2578 | LBOral60LS.PA | LBOral60LS | PA | PP752022 |
| 2578 | LBFecal109LC.PB2 | LBFecal109LC | PB2 | PP752023 |
| 2578 | LBFecal109LC.PB1 | LBFecal109LC | PB1 | PP752024 |
| 2578 | LBFecal109LC.HA | LBFecal109LC | HA | PP752025 |
| 2578 | LBFecal109LC.NS | LBFecal109LC | NS | PP752026 |
| 2578 | LBFecal109LC.NP | LBFecal109LC | NP | PP752027 |
| 2578 | LBFecal109LC.MP | LBFecal109LC | MP | PP752028 |
| 2578 | LBFecal109LC.NA | LBFecal109LC | NA | PP752029 |
| 2578 | LBFecal109LC.PA | LBFecal109LC | PA | PP752030 |
| 2578 | LBFecal22HV.PA | LBFecal22HV | PA | PP752071 |
| 2578 | LBFecal22HV.HA | LBFecal22HV | HA | PP752072 |
| 2578 | LBFecal22HV.MP | LBFecal22HV | MP | PP752073 |
| 2578 | LBFecal22HV.NP | LBFecal22HV | NP | PP752074 |
| 2578 | LBFecal22HV.NA | LBFecal22HV | NA | PP752075 |
| 2578 | LBFecal22HV.PB1 | LBFecal22HV | PB1 | PP752076 |
| 2578 | LBFecal22HV.NS | LBFecal22HV | NS | PP752077 |
| 2578 | LBFecal22HV.PB2 | LBFecal22HV | PB2 | PP752078 |

**Supplemental Table 1.** Summary of real-time reverse transcriptase polymerase chain reaction (qRT-PCR) results for H5 and H7 from poultry live bird market (LBM) samples collected in northern Vietnam from January 10^th^, 2019 to April 26^th^, 2021.

| Sample Types | Number of specimens examined | Number of specimens positive for H5 by qRT-PCR | Percent of specimens positive for H5 by qRT-PCR | Number of specimens positive for H7 by qRT-PCR | Percent of specimens positive for H7 by qRT-PCR |
| --- | --- | --- | --- | --- | --- |
| LBM Poultry Oropharyngeal Swabs | 141 | 24 | 17.0% | 0 | 0.0% |
| LBM Poultry Cage Swabs | 265 | 45 | 17.0% | 0 | 0.0% |
| LBM Bioaerosols | 48 | 14 | 29.2% | 0 | 0.0% |


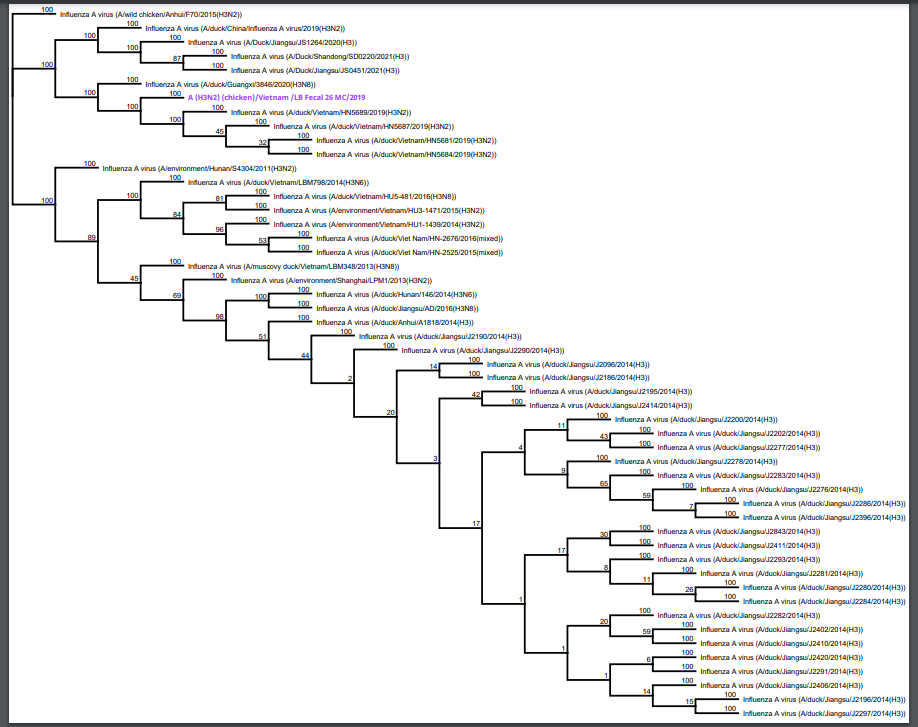


**Supplementary Figure 1:** Neighbor-joining phylogenetic tree of the hemagglutinin (HA) gene segments of the isolated poultry H3N2 virus. Purple = HA sequence from a specimen collected in the Quang Ninh Province, Vietnam; Black = representative H3N2 viruses from GenBank.


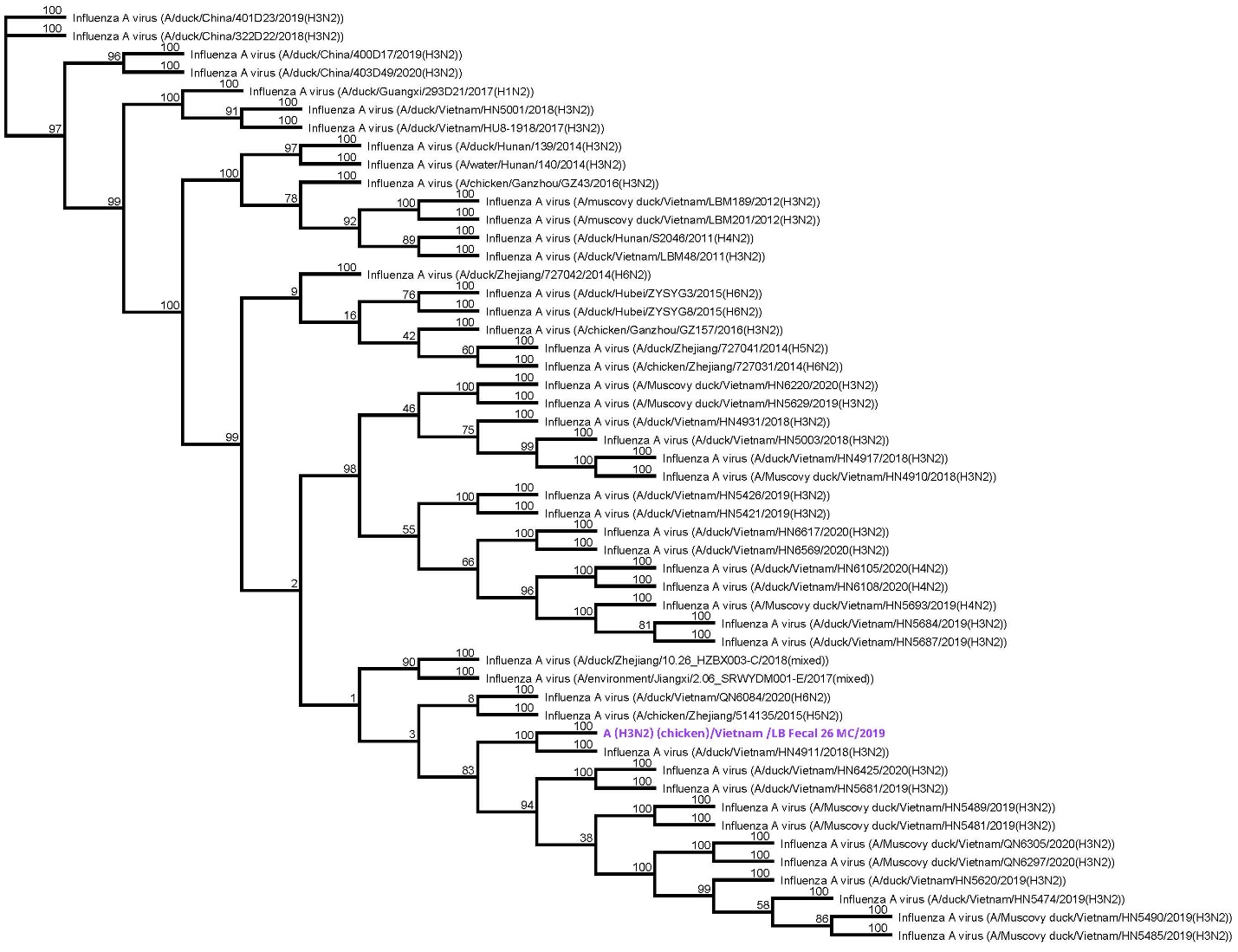


**Supplementary Figure 2:** Neighbor-joining phylogenetic tree of the neuraminidase (NA) gene segment of the isolated poultry H3N2 virus. Purple = NA sequence from a specimen collected in the Quang Ninh Province, Vietnam; Black = representative H3N2 viruses from GenBank.

**
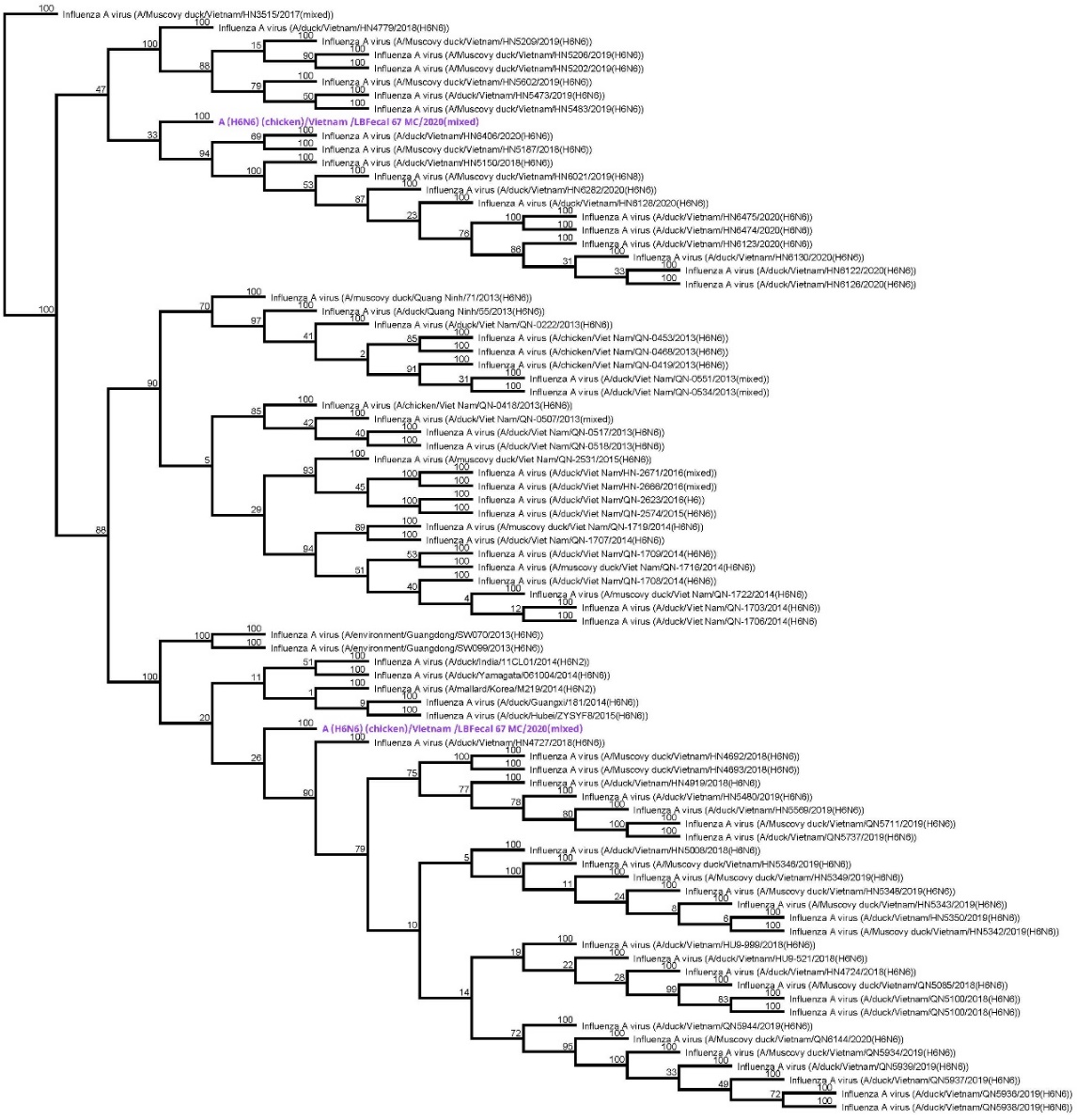
**

**Supplementary Figure 3:** Neighbor-joining phylogenetic tree of the mixed hemagglutinin (HA) gene segments of the isolated poultry H6N6 viruses from one avian sample. Purple = HA sequences from a specimen collected in the Quang Ninh Province, Vietnam; Black = representative H6N6 viruses from GenBank.


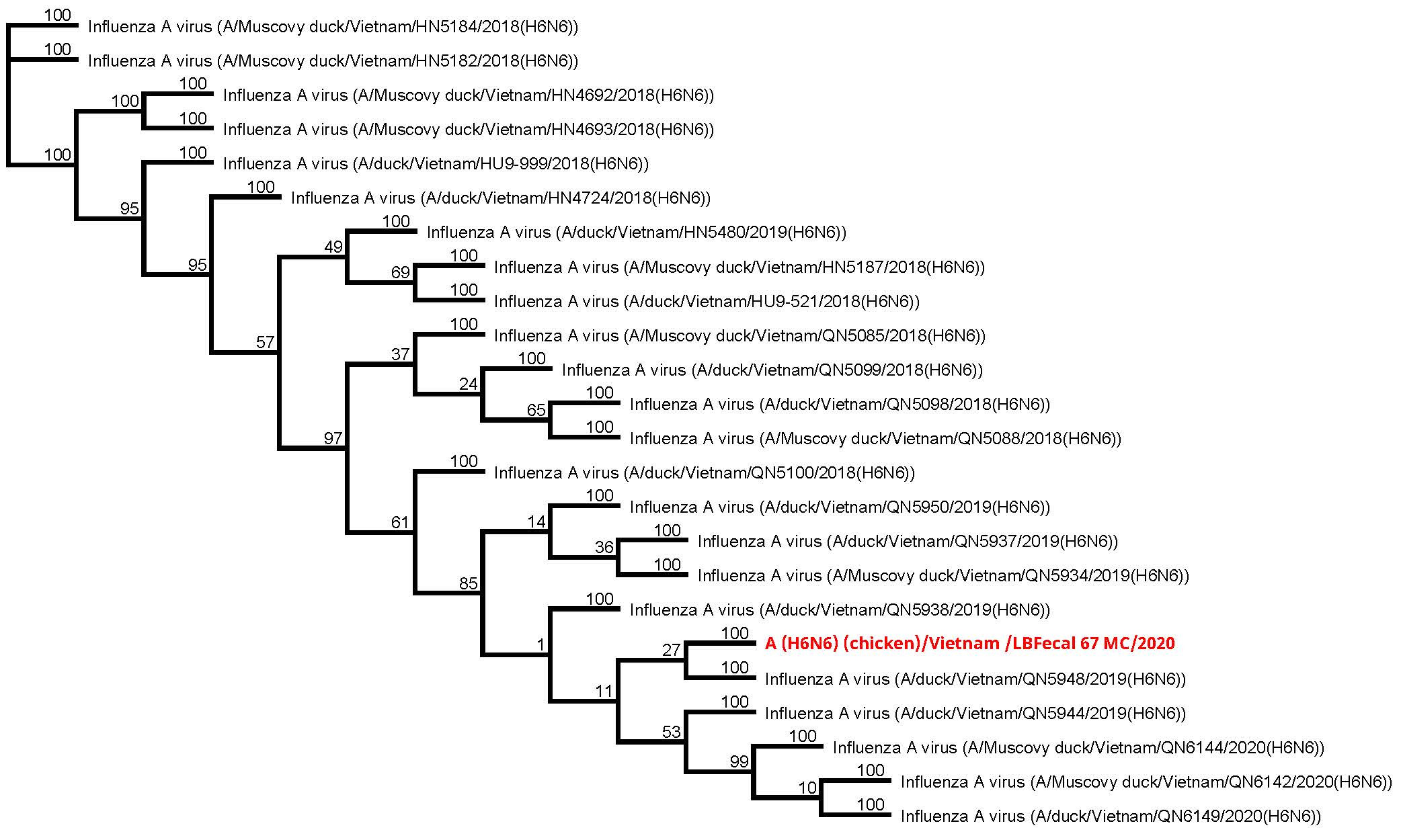


**Supplementary Figure 4:** Neighbor-joining phylogenetic tree of the neuraminidase (NA) gene segment of the isolated poultry H6N6 virus. Red = NA sequences from a specimen collected in the Quang Ninh Province, Vietnam; Black = representative H6N6 viruses from GenBank.

**
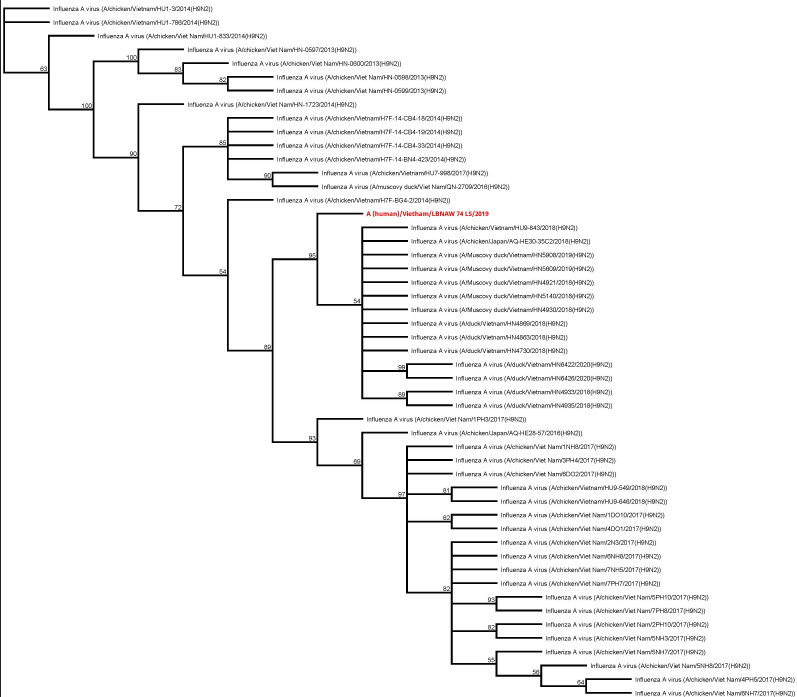
**

**Supplemental Figure 5.** A neighbor-joining phylogenetic tree of a partial PB1 gene segment of the human nasal wash study sample. The partial PB1 gene segment is indicated in red and representative H9N2 viruses from GenBank are black.

**
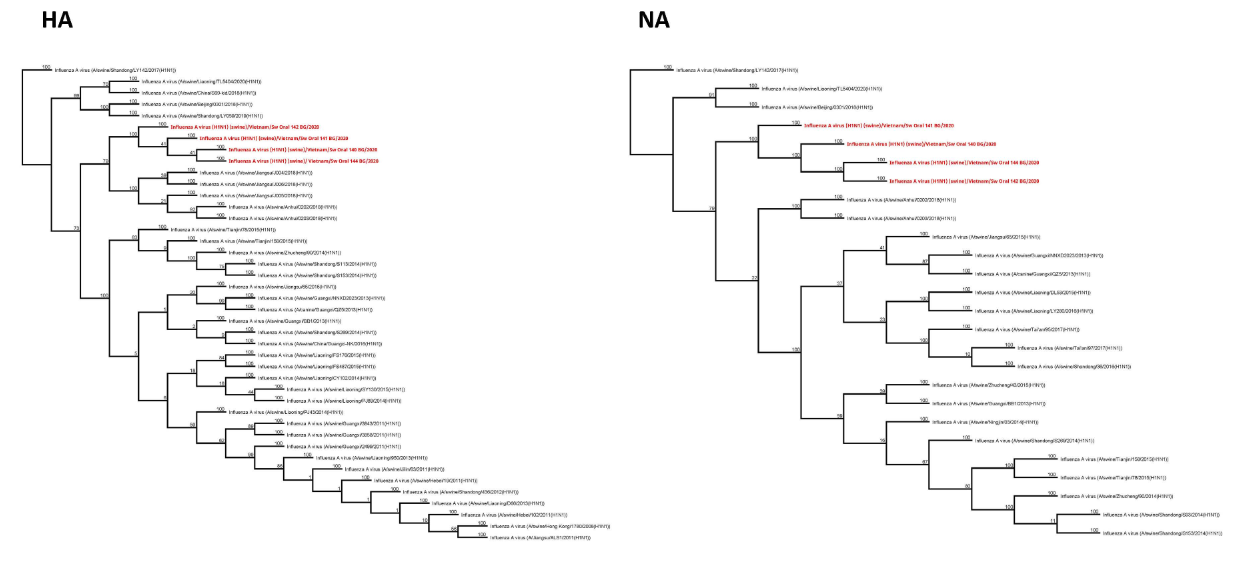
**

**Supplemental Figure 6**. Neighbor-Joining phylogenetic tree of the hemagglutinin (HA) and neuraminidase (NA) genes segments for four swine samples collected in Bac Giang Province, Vietnam. The HA and NA gene segments collected in this study are indicated in red and representative H1N1 viruses from GenBank are black.
