# Supplementary figures and images for: High Prevalence of Highly Pathogenic Avian Influenza: A Virus in Vietnam's Live Bird Markets
Source: Open Forum Infect Dis. 2024 Jul 11;11(7):ofae355. doi: 10.1093/ofid/ofae355 (PMC11250224; doi:10.1093/ofid/ofae355)

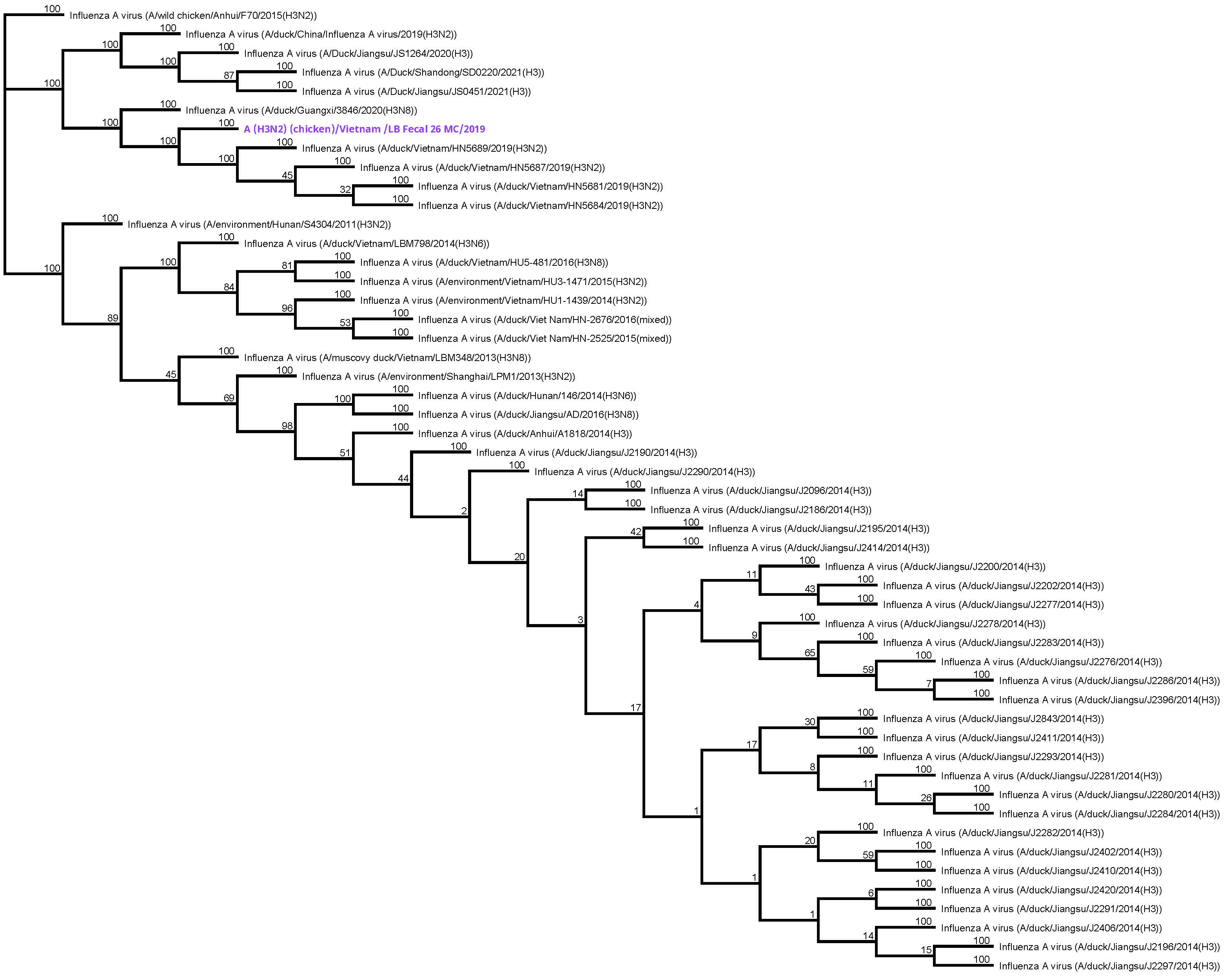

Supplement: ofae355_Supplementary_Data [file ofae355_supplementary_data.zip › Sup Fig 1.tif]

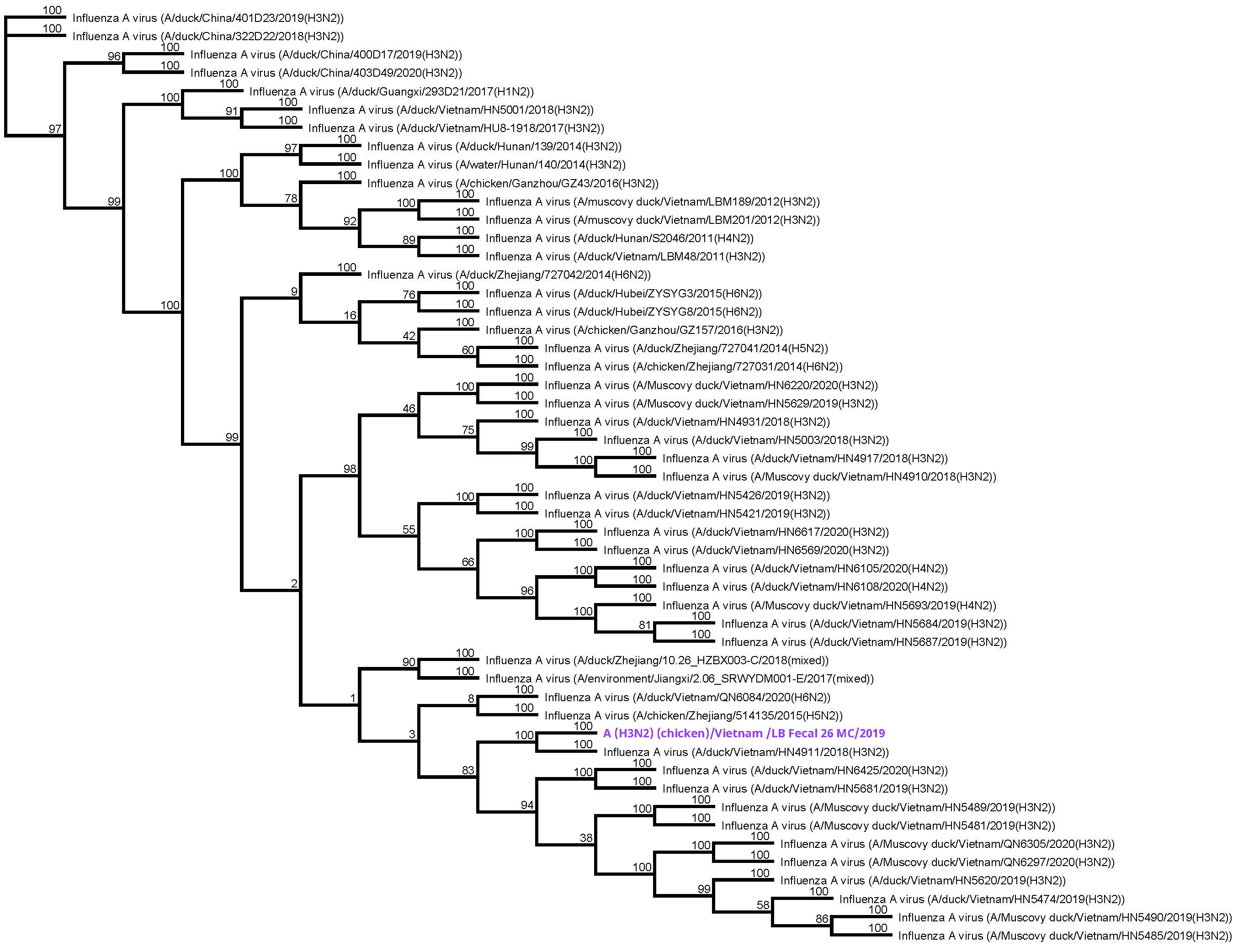

Supplement: ofae355_Supplementary_Data [file ofae355_supplementary_data.zip › Sup Fig 2.tif]

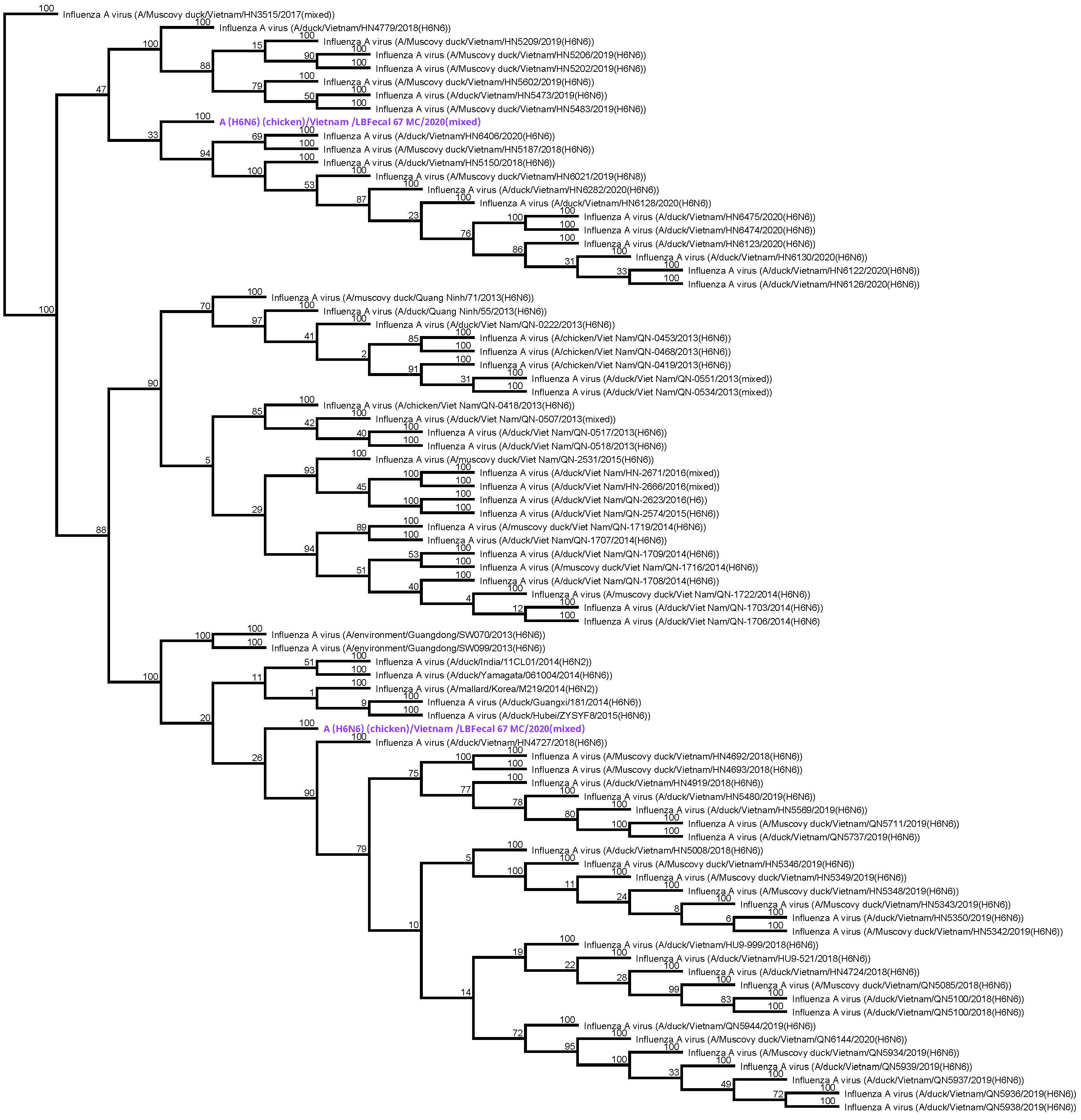

Supplement: ofae355_Supplementary_Data [file ofae355_supplementary_data.zip › Sup Fig 3.tif]

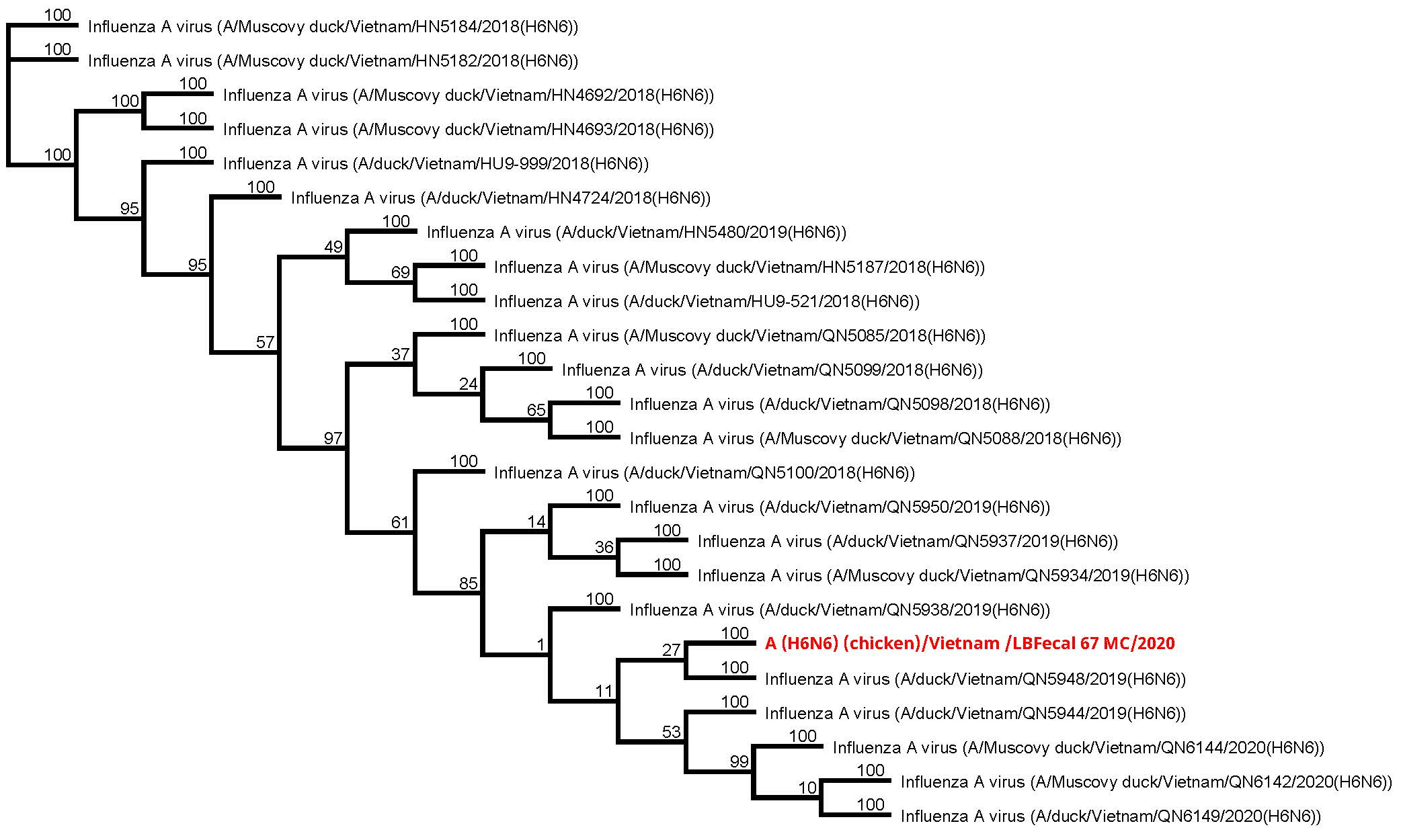

Supplement: ofae355_Supplementary_Data [file ofae355_supplementary_data.zip › Sup Fig 4.tif]

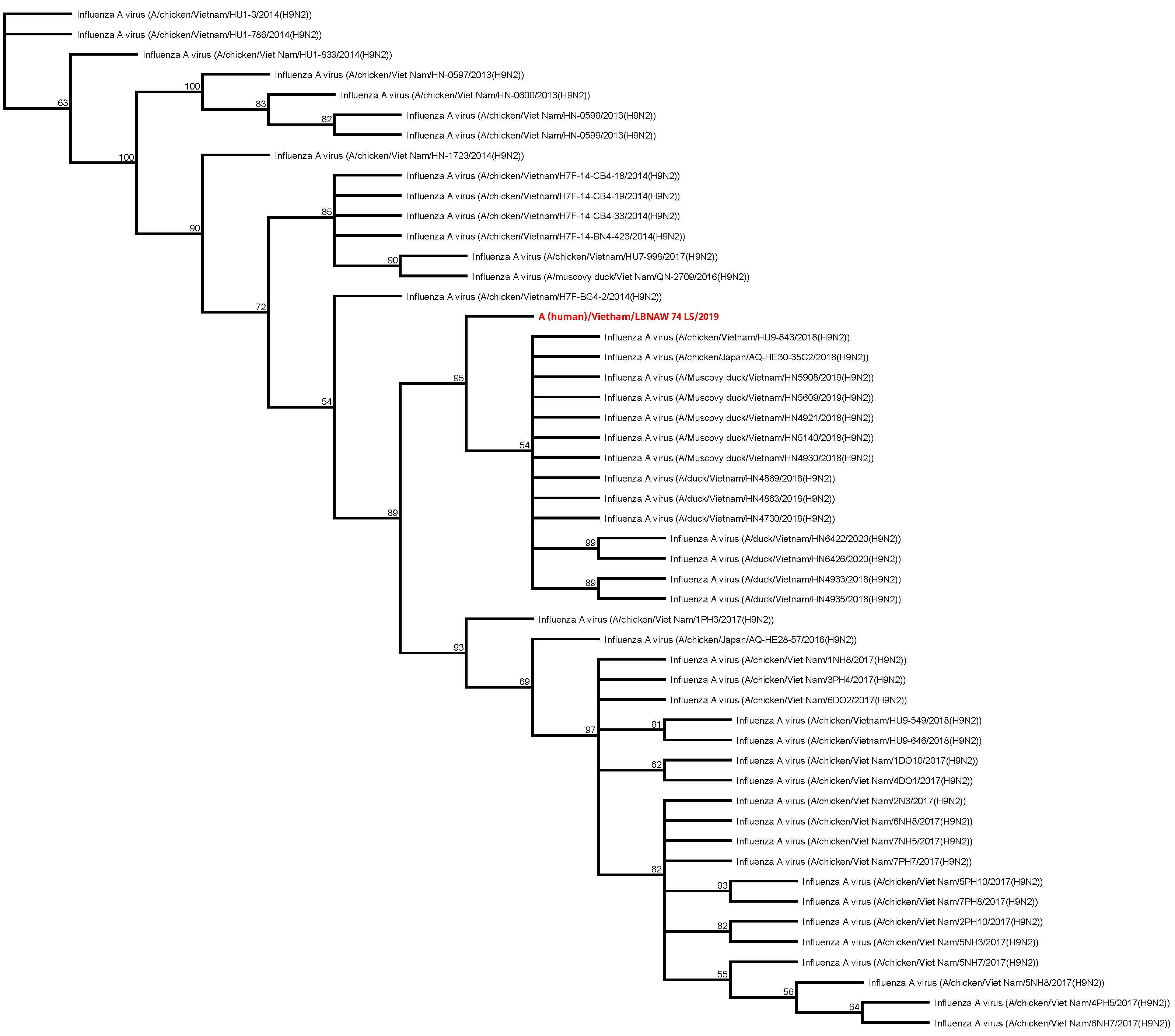

Supplement: ofae355_Supplementary_Data [file ofae355_supplementary_data.zip › Sup Fig 5.tif]

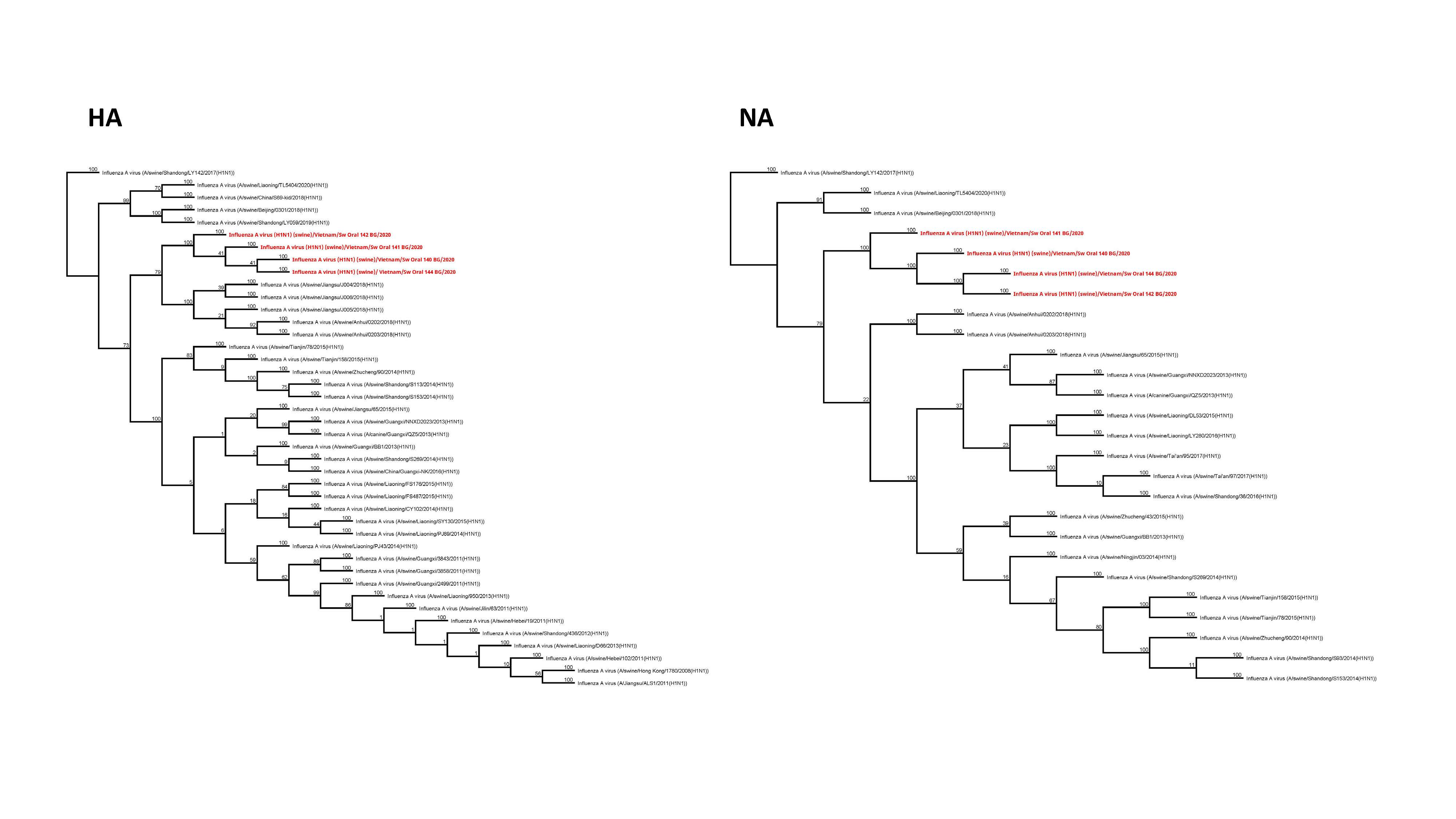

Supplement: ofae355_Supplementary_Data [file ofae355_supplementary_data.zip › Sup Fig 6.tif]
